# Impacts of conventional and organic farming practices on soil and aquatic microbial communities in rice *(Oryza sativa*) agricultural fields in Southern Brazil

**DOI:** 10.1093/femsec/fiag059

**Published:** 2026-06-05

**Authors:** Fernanda B Gazulha, Carla S R Huber, Luciano K Huber, Gabriel Rubensam, Valentina Brocker-Junqueira, Eduardo Moreira da Silva, Joe D Taylor, Laura R P Utz

**Affiliations:** Laboratório de Protistologia, Universidade Católica Pontifícia do Rio Grande do Sul (PUCRS), Porto Alegre, Rio Grande do Sul, CEP 90619-900, Brazil; Laboratório de Protistologia, Universidade Católica Pontifícia do Rio Grande do Sul (PUCRS), Porto Alegre, Rio Grande do Sul, CEP 90619-900, Brazil; Laboratório de Protistologia, Universidade Católica Pontifícia do Rio Grande do Sul (PUCRS), Porto Alegre, Rio Grande do Sul, CEP 90619-900, Brazil; Centro de Pesquisa de Toxicologia e Farmacologia (INTOX), PUCRS, Porto Alegre, Rio Grande do Sul, CEP 90619-900, Brazil; Laboratório de Protistologia, Universidade Católica Pontifícia do Rio Grande do Sul (PUCRS), Porto Alegre, Rio Grande do Sul, CEP 90619-900, Brazil; Laboratório de Microbiologia e Imunologia, PUCRS, Porto Alegre, Rio Grande do Sul, CEP 90619-900, Brazil; Molecular Ecology, UK Centre for Ecology and Hydrology, MacLean Building, Wallingford OX10 8BB, United Kingdom; Laboratório de Protistologia, Universidade Católica Pontifícia do Rio Grande do Sul (PUCRS), Porto Alegre, Rio Grande do Sul, CEP 90619-900, Brazil

**Keywords:** agrochemicals, eDNA, metabarcoding, microbes, protists

## Abstract

Microbial communities play essential roles in agroecosystem functioning, yet the effects of different rice farming practices on their structure and dynamics remain underexplored, particularly across soil and water compartments. This study compared microbial assemblages in organic and conventional rice fields in southern Brazil over a single growing season, based on ten samples collected from two fields. High-throughput sequencing of 16S region V3V4 and 18S region V4 rRNA genes was used to profile bacteria, unicellular eukaryotes, fungi, and metazoa at three time points during rice cultivation. Community composition and diversity differed between farming systems and over time. In soil, bacterial richness was higher in conventional systems at specific time points but showed greater temporal variability. In water, microbial communities in organic systems were generally more diverse and stable, with significantly lower bacterial richness in conventional systems at the initial sampling point (*P <* 0.01). Unicellular eukaryotes and metazoa showed strong farming-system responses, particularly in water, where organic fields supported more diverse assemblages. These findings highlight the influence of farming practices on microbial biodiversity and emphasize the importance of integrated, multi-group approaches for understanding agroecosystem functioning.

## Teaser

Organic and conventional rice farming differently shape soil and water microbial communities, revealing system-specific diversity and temporal patterns across multiple microbial groups using high-throughput sequencing approaches.

## Introduction

Rice (*Oryza sativa* L.) is consumed daily by more than three billion people in Asia and South America (Hansen et al. 2012). As the second most cultivated cereal crop worldwide, it contributes to human nutrition, providing around 21% of the average global per capita energy intake. Rice production in Brazil plays a significant role in the country’s agricultural sector with the southern region being one of the largest areas worldwide in rice production (García et al. 2021).

Rice paddies are among the most intensively managed agricultural ecosystems globally. They are ecologically distinct from most terrestrial agroecosystems, with microbial life thriving in soil (Lopes et al. 2011) and water (Pittol et al. 2018). Microbial communities in these environments are central to nutrient cycling, organic matter decomposition, and interactions with plant roots (Charaslertrangsi et al. 2024). However, how different rice farming systems shape these microbial communities over time remains poorly understood, particularly when accounting for soil and floodwater physicochemical conditions and the diverse range of microbial and mesofaunal groups inhabiting adjacent field habitats.

Organic and conventional rice farming systems differ fundamentally in their management strategies. Organic systems are characterized by the exclusion of synthetic fertilizers, and agrochemicals, relying on organic matter inputs to maintain soil fertility (Oelofse et al. 2011). These practices can promote microbial activity and support a broader diversity of microbial life (Lopes et al. 2011).

Conventional rice farming involves intensive use of mineral fertilizers and agrochemicals, with less emphasis on organic inputs. While these practices can increase crop yields, they may reduce microbial diversity by selecting microbial taxa adapted to high nutrient availability (Sihi et al. 2017). The effects on microorganisms are variable and rely on several factors (Pertile et al. 2020). In addition to shaping bacterial and fungal communities (Suzuki et al. 2019), these inputs may affect higher trophic levels, such as protists and metazoans, which depend on stable and structured food webs.

Within rice agriculture, Glyphosate (N-phospho-nomethylglycine, NPG) is one of the most used non-selective, post-emergent herbicides (Baylis 2000, Rodrigues and Almeida 2005). Despite having been considered a safe chemical, its excessive and inadequate use may contaminate aquatic and terrestrial environments (Kanissery et al. 2019). NPG has an affinity for soil particles, and accumulates on top layers, but studies have shown that it may be transported to lower soil profiles depending on weather conditions following the application (Kanissery et al. 2019, Carretta et al. 2021, Overbeek et al. 2024). In rice crops, microorganisms play a fundamental environmental role in biodegradation of toxic compounds into NPG-degrading metabolites, such as Aminomethyl-phosphonic acid (AMPA), which is degraded mainly by the action of bacteria (Mattos et al. 2002). Although agrochemicals can have impacts on microbial communities, few studies focused on impacts that NPG may have on unicellular eukaryotes and metazoans in these systems (Rosenkranz et al. 2023).

Despite growing interest in agricultural microbiomes, most studies have focused on soil bacteria and fungi (Xu et al. 2022, Yoon Jung et al. 2024) and are restricted to South Asia, with few in other regions (Serbent et al. 2021). This represents a gap in our understanding, since microbial communities in the floodwater can differ substantially in composition and function from those in soil (Liesack et al. 2000). The dynamic of the flooded environment in rice paddies means that microbial communities in soil and water are likely to shift over time, influenced by plant growth, water management, and decomposition of organic residues (Xu et al. 2022).

Microbial eukaryotes and metazoans are rarely included in studies of agricultural microbiomes (Serbent et al. 2021), however, heterotrophic protists play critical roles as bacterial grazers and nutrient recyclers (Asiloglu et al. 2021), while metazoans contribute to soil structure, nutrient turnover, and regulation of microbial populations (Wan et al. 2022). These groups often respond to changes in management, nutrients, and disturbance distinctly from bacteria and fungi, offering a more holistic picture of agroecosystem health and function (George et al. 2019, Köninger et al. 2023).

In this study, we conducted a comparative analysis of microbial communities in the soil and overlying water of two adjacent rice fields in southern Brazil, organically and conventionally managed. Samples were collected at three key points during a single growing season to capture early, mid, and late-season dynamics. Using DNA metabarcoding, we characterized the diversity and taxonomic composition of bacteria, fungi, protists, and metazoans independently in soil and overlying-water samples.

Our aim was to investigate how organic and conventional rice farming systems influence microbial communities in both soil and water over time. Specifically, we asked: (i) how does farming system shape the composition and diversity of microbial taxa in each environment, (ii) how do these communities change across the growing season, and (iii) which microbial groups respond most strongly to management differences? By integrating multiple microbial groups across distinct but interconnected habitats, this study provides a more comprehensive understanding of the ecological effects of rice farming practices and may contribute to efforts to expand and improve sustainable agricultural management practices in commercial rice production.

## Methods

### Sample collection

**Figure 1:**
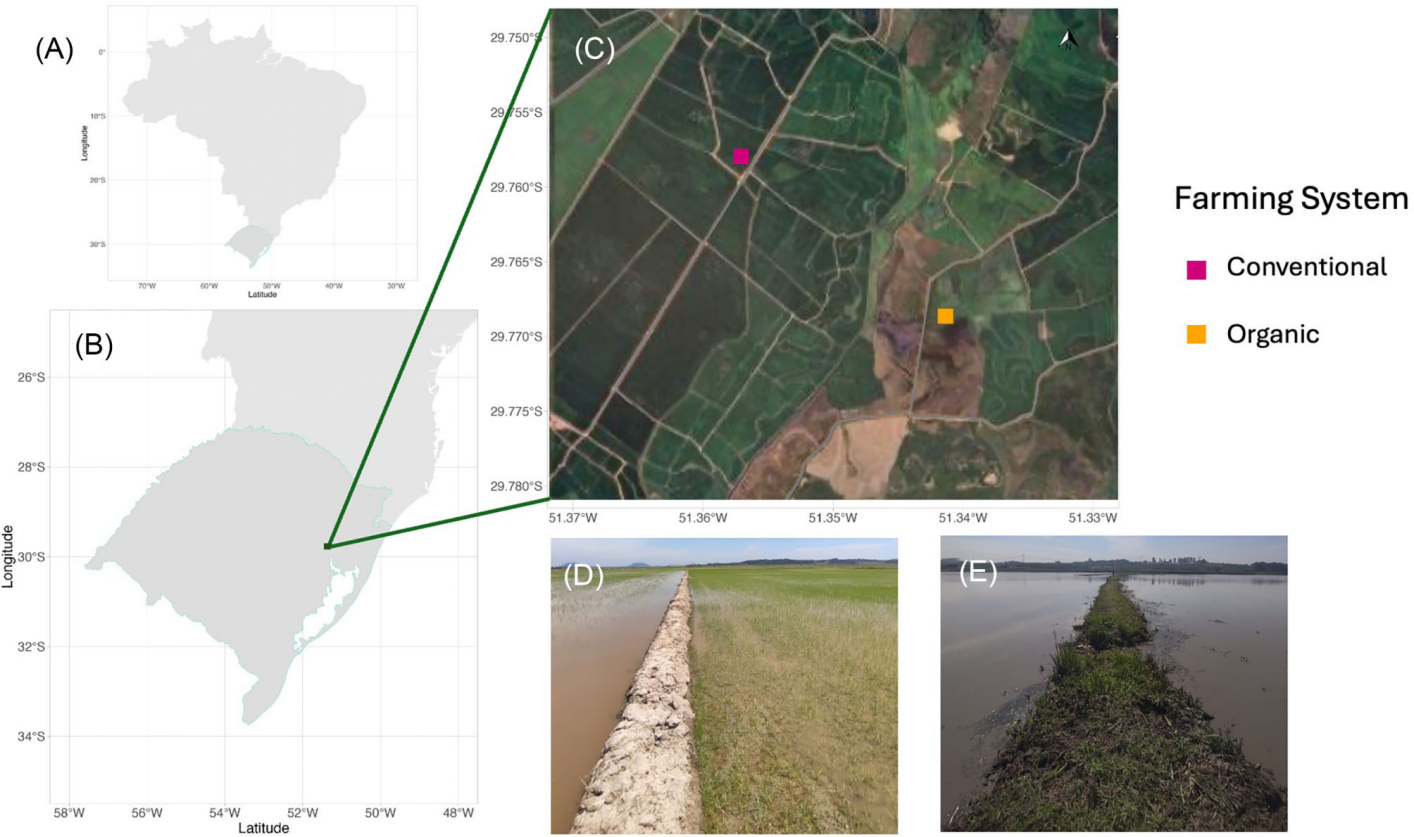
Location of sampling sites. (A) Map of Brazil indicating the southernmost state of Rio Grande do Sul (RS); (B) Map of RS showing the sampling region; (C) Enlarged view of the sampling area with site coordinates: Conventional site (−29.7589160, −51.3589020); Organic site (−29.7714440, −51.3404590); (D) Picture of the conventional site; (E) Picture of the organic site.

Surveyed rice fields were located in Nova Santa Rita, in the metropolitan region of Porto Alegre (Rio Grande do Sul, Brazil) (Fig. 1). Two fields under different types of management were selected within this study area, the selected sites shared key environmental characteristics (soil type, watershed, and regional climate), allowing for a controlled comparison of management effects under similar conditions. However, this means that results should be interpreted as case-specific comparisons rather than fully representative of all organic and conventional rice systems. The fields were 2.2 km apart, shared the same bedrock (Permian sedimentary rock), and lay within the same watershed. Soils in the region are predominantly deep, sandy, well-drained, and acidic, with low base saturation and low natural fertility. They exhibit a high degree of argilluviation and a sequence of A, B, and C horizons. In about 20% of the unit area, hydromorphic soils occur, which are located in basin-like depressions between the elevations. In depressions located between elevations, gleysols are included, occupying less than 10% of the area (https://www.ufsm.br/museus/msrs/unidade-de-solos).

One field, termed “conventional,” used agrochemicals, including glyphosate (RoundUp®), and was managed under conventional practices. In addition to glyphosate, other herbicide such Aminol 2,4-D (2,4-dichlorophenoxyacetic acid) was used, as well as insecticides, and methomyl for pest control. These compounds were not directly quantified in this study, and according to the landowner, in a given growing season, no more than two of these products were typically applied, with the agrochemical prevalent in the system being Glyphosate based. The other, “organic,” field was a certified farm using locally produced compost, prepared in the site comprising a mix of rice husks and straw, swine manure from the property, ash and additional plant residues as tree branches. The compost is normally applied once a year. In addition, this compost may be supplemented with commercially sourced animal manure as poultry or turkey according to availability. Commercial organic fertilizers were never used in the property which characterized a small to medium scale agricultural system.

Soil and water samples were collected from both sites in October and December 2021, and February 2022. Each field was divided into five small plots ∼50 m from each other to capture spatial variability across the area. For each plot, a sampling point was established, and for each sampling point, three samples were collected, spaced ∼5 m apart, and subsequently treated as replicates. In the two subsequent sampling campaigns, samples were taken at the same locations selected during the first sampling campaign, but with possible variation of up to 2 m away from each original location, according to water availability in the field. Rice had been planted at approximately the same time at both sites and sampling was carried out at 5 (Time point 1), 40 (Time point 2), and 121 days (Time point 3) after the rice was planted. In the first sampling campaign seedlings were just emerging from the soil in both fields and each field was completely covered with water. In the second campaign the rice was grown with plants in the organic farm reaching 30–40 cm and in the conventional farm 60–80 cm and much less water was available in the field. In the last sampling campaign, the field was mostly dry with water present only in a channel that ran throughout the field extension, the rice had been harvest and the field was covered by rice straw. The amount of water varied throughout the rice cultivation period. At the beginning of the cycle, the water column was ∼20 cm. Closer to the end of the cultivation period, the water depth decreased to around 10 cm, with some areas already presenting dry patches. Water samples were consistently collected from the surface layer to ensure comparability across sites and times. At each timepoint, 100 g of surface soil was collected using a sterilized spoon, stored in 50 ml falcon tubes, kept on ice for transportation, and frozen at –20°C until DNA extraction. A total of 200 ml of water was collected in sterile plastic flasks, transported in a similar manner, and stored at 4°C until filtration. For DNA analysis, 50 ml of each water sample was filtered through a 0.22 µm cellulose acetate membrane using an electric pump. Filters were stored in Eppendorf tubes at –20°C. Filtrates were also frozen at –20°C for later analysis of NPG and AMPA concentrations.

### Quantification of NPG and AMPA

NPG and AMPA were quantified in soil and water samples by liquid chromatography coupled with mass spectrometry (LC-MS/MS) employing the Agilent 1290 Infinity system with a 6460 Spectrometer (Agilent Technologies, Santa Clara, CA, USA). The methods used for the analyses followed Stefani et. al. (2020), with modifications. Soil (2.5 g) and water (1.0 ml) samples were treated with 5% ammonium hydroxide, vortexed for 30 min at 70 RPM at room temperature. After that, a strip of filter paper (44-µm pore) was added to each sample, with 10 mm of the material submerged, standing for 20 min to allow the liquid phase migrate through the paper. The clear upper portion was cut and transferred to a 1.5 ml centrifuge tube with a holder. After centrifugation at 14 000 RPM, at 4°C, for 20 min, 20 µl of the extract was collected for analysis. The determination and quantification of NPG and AMPA were performed on a Hypercarb C18 chromatographic (Thermo Scientific, USA) column, using a mobile phase consisting of 5 mM ammonium acetate pH 10 and acetonitrile in gradient mode. Analyses were performed in MRM mode, considering m/z 168–81 and 168–62.8 for NPG, and m/z 110–79 and 110–62.8 for AMPA quantification and confirmation, respectively. Quantification was performed by external standardization, and the calibration curve was constructed in the range of 0.1 to 3.0 mg/l. Results were expressed in mg/kg for NPG and AMPA in soil samples and mg/l for water samples.

### DNA extraction, PCR, and Sequencing

DNA was extracted from soil samples (0.25 g) and from water sample filters using the DNeasy PowerSoil Pro (QIAGEN) kit according to the manufacturer’s instructions. DNA was quantified using a NanoDrop Lite Spectrophotometer (Thermo Fisher Scientific). DNA was sent to a commercial sequencing facility for PCR and sequencing (IMR, Canada). Library preparation was a “one-step” reaction and PCR primers contained indices and Illumina adapters. The 16S rRNA of Bacteria (regions V3–V4) was amplified using primers 341F 5′-CCTACGGGNGGCWGCAG-3′ and 806RB 5′- GGACTACNVGGGTWTCTAAT-3′ (Klindworth et al. 2013). The conditions for amplification were: initial activation at 94°C for 5 min, 30 cycles of 30 s at 94°C and 30 s at 43°C, followed by a 72°C extension for 1 min 30 s and 7 min at 72°C. The 18S rRNA of eukaryotes (V4 region) was amplified using primers E527F 5′-CYGCGGTAATTCCAGCT-3′ and E1009R 5′-AYGGTATCTRATCRTCTTYG-3′ (Comeau et al. 2011). The conditions for amplification were: initial activation at 94°C for 5 min, 35 cycles of 30 s at 94°C and 45 s at 55°C, followed by a 72°C extension for 90 s. Sequencing was performed using the Illumina MiSeq platform at the Integrated Microbiome Resource laboratory using a 2 × 300 bp v3 sequencing kit (IMR, Canada).

### Sequence processing and classification

Demultiplexed sequences with adapters removed, were processed in R (version 4.3.0) (Callahan et al. 2016) primer sequences were removed using cutadapt (Martin 2011) and the DADA2 package according to the following steps: primer removal, quality filtering (maxEE = 3; truncLen: R1 = 280, R2 = 250), forming amplicon sequence variants (ASVs) merging, chimera removal and taxonomic assignments using SILVA (16S version 138.2; Quast et al. 2012) and PR2 18S (Version 5.0; Guillou et al. 2012) databases. Sequences that could not be assigned to these databases or specific ASVs of interest were manually checked using a BLAST search against the full NCBI database (https://blast.ncbi.nlm.nih.gov/Blast.cgi). From the Bacteria dataset, mitochondria and chloroplasts sequences were removed. Archaea sequences were also excluded from downstream analyses. The 341F–806RB primer set is optimized for Bacteria and provides limited archaeal coverage, resulting in their underrepresentation. Archaea reads comprised <0.1% of total sequences and were inconsistently detected across replicates, preventing robust statistical analysis. For the Eukaryote dataset embryophytes (mainly rice) and sequences matching Chordata were removed. Across both datasets ASVs with less than four reads were excluded from the analysis. The ASV table was then rarefied for bacteria to 4000 reads per sample; while eukaryotic ASVs were divided into separate taxonomic groups and rarified to 1209 reads per sample for unicellular eukaryotes, 500 for fungi and 400 for Metazoa.

### Statistical analyses

Data was imported in R and structured using the R package phyloseq (McMurdie and Holmes 2013), which enabled integration of amplicon sequence variant (ASV) tables, taxonomic assignments, sample metadata, and phylogenetic information. For statistical analyses a normal distribution was evaluated using the Asymptotic one-sample Kolmogorov–Smirnov and the Anderson–Darling Normality tests (package nortest). Taxonomy plots, alpha diversity metrics, specifically observed richness and Shannon diversity were calculated and visualized using the microeco package (Liu et al. 2021)). Beta diversity was assessed by calculating Bray–Curtis dissimilarities, followed by ordination via Principal Coordinates Analysis (PCoA) using microeco. All means are presented as mean ± standard error. Statistical differences of taxonomic relative abundance of the various groups in each farming system at each time point were assessed using Kruskal–Wallis test followed by a Dunn’s post hoc analysis. Alpha diversity differences among groups were assessed using the Wilcoxon signed-rank tests to find pairwise differences, as implemented in vegan (Oksanen et al. 2024) and FSA packages. To assess differences in overall community composition across farming systems and time, we performed two-way permutational multivariate analysis of variance (PERMANOVA) using the adonis2 function in the vegan package in R. Bray–Curtis dissimilarities were calculated from rarefied abundance data, and PERMANOVA was applied with Farming, Time, and their interaction as fixed effects. To account for repeated sampling at the same locations across time points, permutations were constrained within unique site identifiers using the strata argument. The model was run with 999 permutations.

To examine changes in alpha diversity and taxon-specific relative abundances over time and between farming systems, we fitted linear mixed-effects models using the lme function from the nlme package in R. Farming, Time, and their interaction were included as fixed effects, and a random intercept was included for each SiteID to account for repeated measurements at the same sampling locations across time. Model assumptions were assessed via inspection of residual plots. Shannon diversity and class-level relative abundances were used as response variables in separate models

## Results

### Concentrations of NPG and AMPA in soil and water

NPG and AMPA were detected in soil samples from conventional farming plots at all timepoints (Table 1). NPG concentrations ranged from 0.68 ± 0.16 mg/kg to 1.87 ± 1.32 mg/kg, while AMPA concentrations ranged from 0.61 ± 0.12 mg/kg to 0.66 ± 0.47 mg/kg, with no significant difference between timepoints (Kruskal–Wallis test, *P* > 0.05) (Table 1). The relatively high variability observed in some measurements likely reflects spatial heterogeneity in herbicide distribution and environmental conditions within the sampled plots. In contrast, soil samples from organic plots consistently contained levels below 0.1 mg/kg for both compounds, with occasional detections of glyphosate at 0.35 mg/kg (day 5) and 0.15 mg/kg (day 121); AMPA remained undetectable in all organic samples (Table 1).

**Table 1:** Mean glyphosate (NPG) and AMPA concentration ± standard error in soil (mg/kg) and water (mg/l) samples collected from conventional and organic agricultural fields growing rice. Samples were taken across three time points corresponding to 5 days after NPG application in the conventional agriculture, 40 days after application and 121 days after application. Concentrations were measured using LC-MS/MS, limits of detection were <0.1 mg/kg or mg/l.

	Soil		Water	
Farming and time	Glyphosate (NPG) mg/kg	AMPA mg/kg	Glyphosate (NPG) mg/l	AMPA mg/l
Conventional 1 (5days)	1.08 ± 0.2	0.61 ± 0.12	5.88 ± 3.53 (n = 3) < 0.1 (n = 2)	All < 0.1
Conventional 2 (40days)	1.87 ± 1.32	0.66 ± 0.47	All < 0.1	All < 0.1
Conventional 3 (121days)	0.68 ± 0.16	0.62 ± 0.21	< 0.1–0.19	All < 0.1
Organic 1 (5days)	< 0.1 (n = 4), 0.35	All < 0.1	All < 0.1	All < 0.1
Organic 2 (40days)	All < 0.1	All < 0.1	All < 0.1	All < 0.1
Organic 3 (121days)	< 0.1 (n = 4), 0.15	All < 0.1	All < 0.1	All < 0.1

In water samples from conventional plots, glyphosate was detected on day 5 (5.88 ± 3.53 mg/l) and between <0.1–0.19 mg/l on day 121, while no AMPA was detected at any time point. All water samples from organic plots were below detection limits for both NPG and AMPA throughout the study (Table 1).

### Sequencing run metrics and overall dataset taxonomic composition

After quality filtering, chimera identification and filtering for ASVs with less than four reads in size, bacteria dataset contained 665 650 sequences and 10 444 ASVs, with a mean number of 11 678 ± 716 sequences per sample in a total of 57 successfully sequenced samples. Archaea made up <0.1% of the total prokaryotic reads, with inconsistent read depth per sample and therefore were not analyzed further. The bacteria dataset contained 40 different phyla and 106 different classes. The total eukaryotic 18S rRNA dataset contained 9021 ASVs in 1 432 263 sequences in 52 successfully sequenced samples. Of this, the unicellular eukaryote dataset contained 732 438 total sequences and 6 805 ASVs with a mean of 14 085 ± 2250 sequences per sample, across 225 different families. Fungi dataset contained 1467 ASVs in 240 396 sequences with a mean of 4623 ± 808.81 sequences per sample across 13 phyla and 42 classes. Metazoa dataset contained 673 ASVs in 238 119 sequences with a mean of 4481.70 ± 805.67 sequences per sample.

### Differences in richness and diversity between farming system and time points in soil and water samples

**Figure 2:**
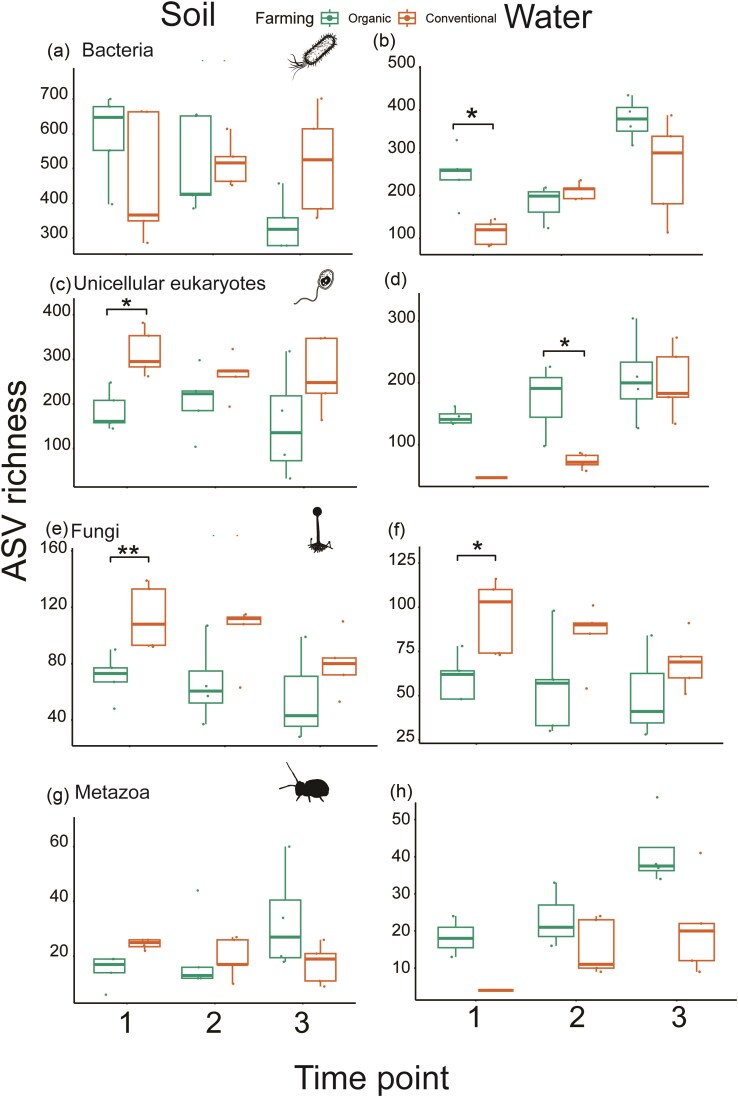
ASV richness for bacteria (a, b), unicellular eukaryotes (c, d), fungi (e, f), and Metazoa (g, h), both for organic and conventional systems at sampling events 1, 2, and 3, respectively. Boxplots display the distribution of ASV richness across farming systems and time points. The boxes represent the interquartile range (IQR), with the horizontal line indicating the median. Whiskers extend to values within 1.5 times the IQR, while points beyond the whiskers denote outliers. Asterisks indicate significant differences (* *P <* 0.05, ** *P <* 0.01; Wilcoxon signed-rank test). Note that *y*-axis scales differ between panels.

**Figure 3:**
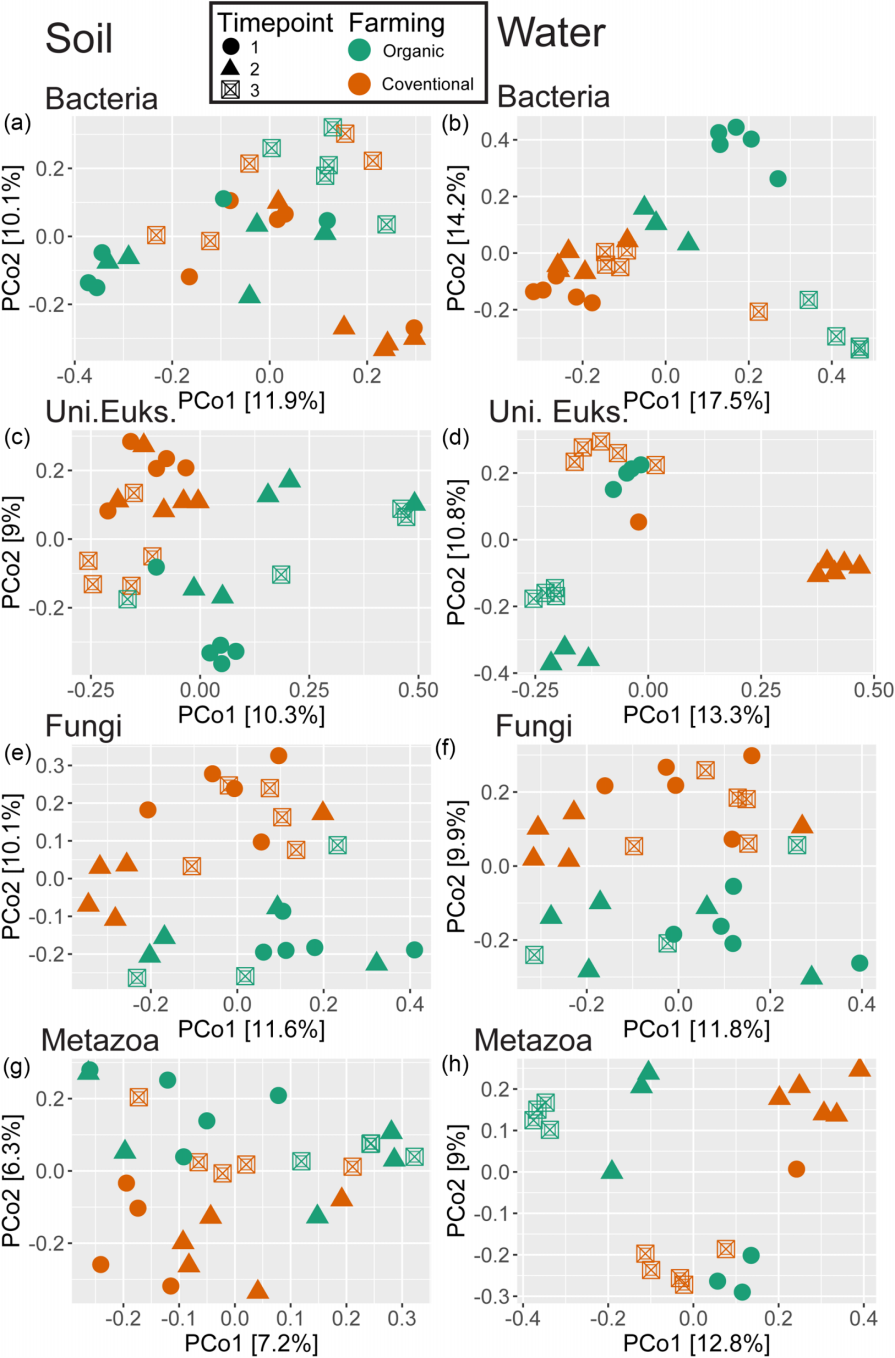
Principal coordinate analysis based on Bray–Curtis dissimilarity matrices of ASV community composition in soil and water samples from organic and conventional farm at three time points for bacteria (a, b), unicellular eukaryotes (c, d), fungi (e, f), and Jaccard similarity matrices for Metazoa both for organic and conventional farming systems at sampling time points 1(circle), 2 (triangle), and 3 (crossed square) respectively.

Richness and diversity patterns differed between farming systems and across sampling times in both soil and water (Fig. 2). Bacterial ASV richness in soil (Fig. 2a) showed no significant differences between farming systems or across time points, though variability was high among individual samples. In water (Fig. 2b), bacterial richness was significantly lower at the conventional system compared to the organic system at time 1 (Wilcoxon, *P <* 0.001) and increased significantly by time 3 compared to time 1 (Wilcoxon, *P <* 0.001). Bacterial diversity showed no significant differences in soil but was significantly lower in conventional water compared to organic at time 1 (Wilcoxon, *P* < 0.001). Unicellular eukaryote richness in soil (Fig. 2c) was significantly higher in conventional farming at time 1 (Wilcoxon, *P <* 0.0001), with similar but non-significant trends at times 2 and 3. In water (Fig. 2d), richness was significantly higher in organic farming at time 2 (Wilcoxon, *P <* 0.001), with no significant differences at other times. Diversity was significantly lower in conventional water compared to organic at times 2 and 3 (Wilcoxon, *P <* 0.01; Supplementary Fig. 1S). Fungal richness in soil (Fig. 2e) was significantly higher in conventional farming at time 1 (Wilcoxon, *P <* 0.000001), with non-significant trends at times 2 and 3. In water (Fig. 2f), fungi richness was also significantly higher in conventional farming at time 1 (Wilcoxon, *P <* 0.001), with similar but non-significant trends at later times. Diversity showed a non-significant trend of higher values in conventional farming for both soil and water. Metazoan communities showed no clear patterns or significant differences in richness (Fig. 3g and h) or diversity (Supplementary Fig. 1S) between farming systems in either soil or water.

### Differences in community composition between conventional and organic soils and overlying water

Community composition differed significantly between organic and conventional farming systems and across sampling times in both soil and water (Fig. 3). Bacterial communities in soil (Fig. 3a) varied between farming systems at times 1 (PERMANOVA, *P =* 0.021) and 2 (PERMANOVA, *P =* 0.019), with temporal shifts observed in both conventional (time 1 vs 2, PERMANOVA, *P =* 0.040; 2 vs 3, *P =* 0.025) and organic soils (time 1 vs 3, PERMANOVA, *P =* 0.010; 2 vs 3, PERMANOVA, *P =* 0.014). In water (Fig. 3b), bacterial communities consistently differed between systems at all three times (time 1, PERMANOVA, *P =* 0.014; time 2, *P =* 0.023; time 3, *P =* 0.013), with temporal variations within both systems (conventional: times 1 vs 2, PERMANOVA, *P =* 0.005; 1 vs 3, *P =* 0.011; 2 vs 3, *P =* 0.024; organic: 1 vs 2, *P =* 0.019; 1 vs 3, *P =* 0.008; 2 vs 3, *P =* 0.027). Unicellular eukaryote communities in soil (Fig. 3c) differed between farming systems at all times (time 1, PERMANOVA, *P =* 0.010; time 2, *P =* 0.018; time 3, *P =* 0.037), with significant temporal shifts within conventional (times 1 vs 2, PERMANOVA, *P =* 0.009; 1 vs 3, *P =* 0.008; 2 vs 3, *P =* 0.032) and organic soils (times 1 vs 2, PERMANOVA, *P =* 0.035; 1 vs 3, *P =* 0.010). In water (Fig. 3d), differences between farming systems occurred at times 2 (PERMANOVA, *P =* 0.020) and 3 (PERMANOVA, *P =* 0.016), and temporal differences were found within conventional (time 2 vs 3, PERMANOVA, *P =* 0.007) and organic waters (times 1 vs 2, PERMANOVA, *P =* 0.034; 1 vs 3, *P =* 0.033; 2 vs 3, *P =* 0.028). Soil fungal communities (Fig. 3e) differed significantly between farming systems at times 1 (PERMANOVA, *P =* 0.008) and 2 (PERMANOVA, *P =* 0.020), with temporal variations within conventional (times 1 vs 2, PERMANOVA, *P =* 0.028; 1 vs 3, *P =* 0.023; 2 vs 3, *P =* 0.016) and organic soils (time 1 vs 3, PERMANOVA, *P =* 0.019). Fungal water communities (Fig. 3f) showed no significance over time within farming type but there were significant differences between systems at each time points (PERMANOVA, *P <* 0.05). Metazoan communities in soil (Fig. 3g) varied between systems at times 1 (PERMANOVA, *P =* 0.018) and 3 (PERMANOVA, *P =* 0.014), with temporal changes within conventional (time 1 vs 3, PERMANOVA, *P =* 0.008) and organic soils (time 1 vs 3, PERMANOVA, *P =* 0.032). In water (Fig. 3h), metazoan communities differed between systems at times 2 (PERMANOVA, *P =* 0.017) and 3 (PERMANOVA, *P =* 0.010), with temporal differences within conventional (time 2 vs 3, PERMANOVA, *P =* 0.013) and organic waters (times 1 vs 3, PERMANOVA, *P =* 0.025; 2 vs 3, PERMANOVA, *P =* 0.030).

### Impact of farming system on bacteria taxonomic composition

#### Bacteria

**Figure 4:**
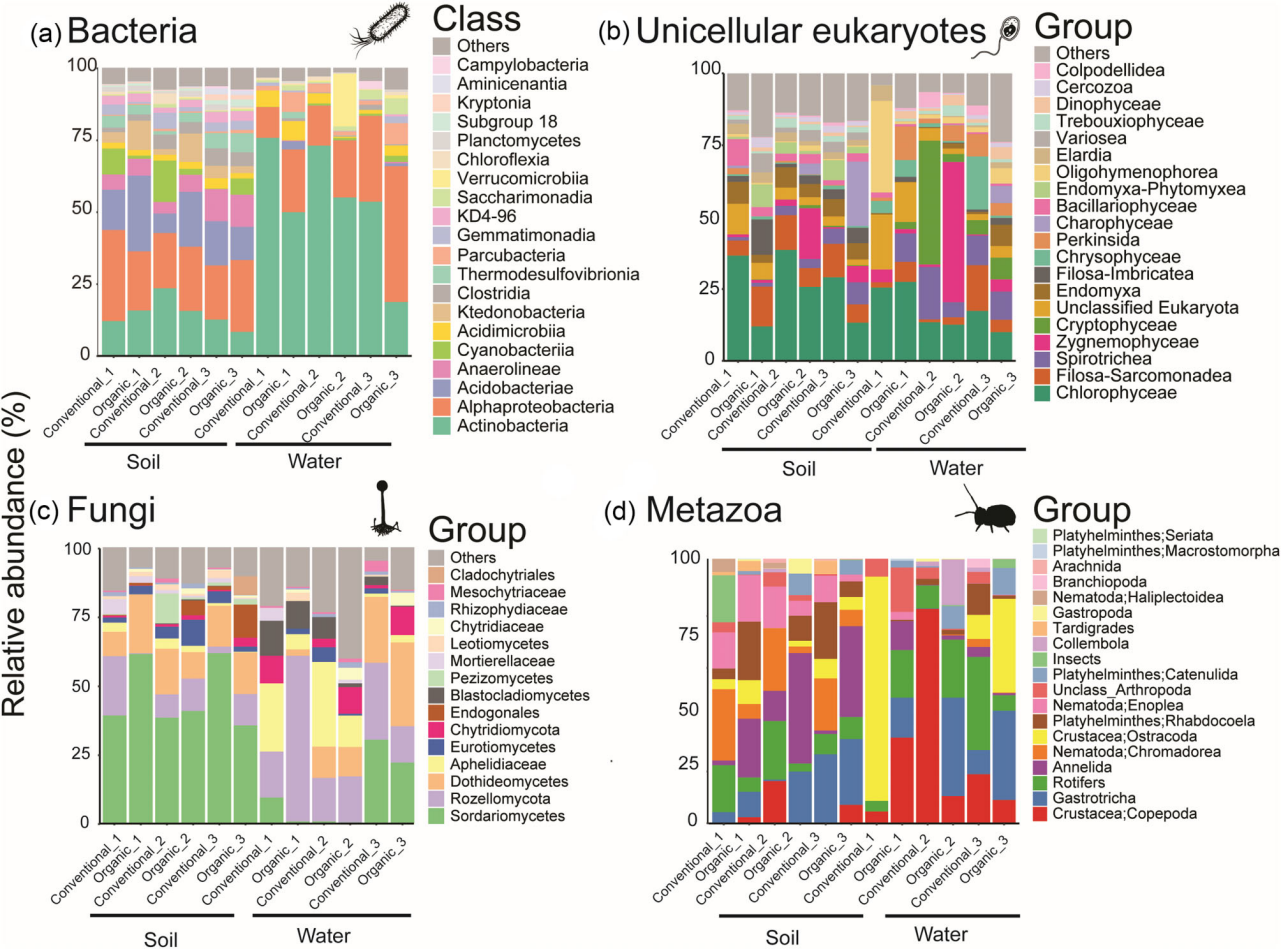
Mean relative abundance (n = 5) of (a) bacteria in 16S rRNA gene metabarcoding libraries, (b) unicellular eukaryotes, (c) fungi and (d) Metazoa in 18S rRNA gene metabarcoding libraries, in soil and overlying water samples from rice agriculture.

In soil, Alphaproteobacteria, Acidobacteriae, and Actinobacteria were the most abundant classes (Fig. 4a). At time point 1, Alphaproteobacteria was more abundant in conventional soils (30.5% ± 4.3%) than in organic (20.5% ± 2.1%) (*P =* 0.047), but no differences were observed at time points 2 or 3 (Fig. 4a). Although their relative abundances in soil varied slightly over time in both systems, Acidobacteriae were consistently more abundant in organic soils (19.0% ± 1.7%) than in conventional soils across all time points (Fig. 4a) (*P =* 0.016). Actinobacteria were highly abundant in both systems, peaking in conventional soils at time point 2 and in organic soils at time point 3 (Fig. 4a). Although differences in Actinobacteria relative abundance between farming systems were not significant at any single time point (*P* > 0.1), there was significant temporal variation. In conventional soils, abundance increased from time 1 to 2 (*P =* 0.033), then declined by time 3 (*P =* 0.016). Organic soils showed a similar temporal pattern, with an increase from time 1 to 3 (*P =* 0.014; Fig. 4a).

In water samples, communities were dominated by Alphaproteobacteria and Actinobacteria (Fig. 4a). Actinobacteria accounted for 76% ± 4% in conventional water at time point 1 and 58% ± 5% in organic water at the same time point. A similar temporal pattern was observed for Actinobacteria in conventional water at time point 2 (Fig. 4a) compared to organic water. Alphaproteobacteria and Actinobacteria abundances in conventional systems rose from time 1 to 2 (*P =* 0.041), then remained stable. In organic systems, Actinobacteria increased from time 1 to 3 (*P =* 0.049).

#### Unicellular eukaryotes

In soil samples, Chlorophyceae and Endomyxa were the most abundant protist groups, especially in conventional systems (Fig. 4b). Filosa (Sarcomonadea and Imbricatea), Gregarinomorphea, and Zygnemophyceae also contributed prominently to the community in organic soils. At time point 1, the Filosa–Sarcomonadea were more abundant in organic soils (13.9% ± 2.5) than in conventional soils (5.2% ± 1.6; *P =* 0.0283; Fig. 4b). A similar pattern was observed for Filosa-Imbricatea, which was also more abundant in organic soils (12.3% ± 1.9) than in conventional soils (2.0% ± 1.4; *P =* 0.0163; Fig. 4b). Gregarinomorphea was also more abundant (*P =* 0.0283; Fig. 4b) in organic soil (4.8% ± 1.7) than in conventional (0.9% ± 0.6; *P =* 0.0283; Fig. 4b), similar to Zygnemophyceae in organic (5.7% ± 1.1) and conventional (0.5% ± 0.2; *P =* 0.0143). In soil, Cryptophyceae peaked in traditional systems at time 2 (2.3% vs. 0.1%, *p =* 0.0455), and Chlorophyceae at time 1 (36.6% vs. 12.0%, *P =* 0.009) and 3 (29.1% vs. 13.2%, *P =* 0.0143; Fig. 4b).

In water samples, Zygnemophyceae dominated the organic systems, particularly at time 2 (Fig. 4b). A pronounced peak in Cryptophyceae was observed in conventional systems at the same time point, reaching 43.1% compared to 2.8% in organic samples (*P =* 0.0253; Fig. 4b). Chrysophyceae peaked in conventional systems at time 3, reaching 18.5% compared to 0.8% in organic systems (*P =* 0.0143; Fig. 4b). Colpodellidea and Endomyxa also had substantial representation, with clear differences between farming systems in their relative abundances over time (Fig. 4b).

Filosa–Thecofilosea and Filosa–Sarcomonadea were more prevalent in organic water at time 3, with significant difference (*P* = 0.0249; Fig. 4b) of the former. Colpodellidea and Chrysophyceae were more abundant in conventional water at time 3 (*P =* 0.0143 and *P =* 0.0143, respectively) while Oligohymenophorea had a peak in a single sample in the conventional water at time 1 (Fig. 4b).

#### Fungi

In soil samples, fungal communities were dominated by members of the Sordariomycetes and Dothideomycetes across both farming systems and time points (Fig. 4c). Sordariomycetes consistently had the highest relative abundances, particularly in organic soils. Fungal community composition in soil varied over time and differed between samples from organic and conventional rice farming systems. At time 1, Rozellomycota was significantly more abundant in conventional soils (21.5% ± 6.4) than in organic soils (0.3% ± 0.2; *P =* 0.008; Fig. 4c). but this difference was not maintained at later time points, and a shift in abundance in organic soils was observed at times 2 and 3 (Fig. 4c) Sordariomycetes dominated the community at all soil samples, particularly in organic soils at time 1 (Fig. 4c). Dothideomycetes were abundant, with a trend to higher abundance in conventional soils than in organic soils at time 2 with similar levels at time 3 (Fig. 4c). Pezizomycotina was more abundant in organic soils at time 1 (*P =* 0.0374; Fig. 4c), while Endogonales appeared only in organic soils at times 2 and 3 (Fig. 4c).

In water samples, Rozellomycota was the most dominant group, particularly in organic systems at time 1. Sordariomycetes, Aphelidiaceae, and Chytridiaceae also contributed substantially to the community composition, with notable differences in representation between organic and conventional systems (Fig. 4c).

Rozellomycota dominated organic water samples at times 1 and 2, but at time 3 peaked in the conventional system (Fig. 4c). Sordariomycetes was more abundant in conventional water samples at all time points (39%–63%), while Chytridiaceae was enriched in organic water samples at time 3 (4.7% ± 1.4 vs. 0.0%; *P =* 0.0073; Fig. 4c). Eurotiomycetes was significantly more abundant in conventional water samples only at time 3 (1.9% ± 0.9 vs. 0.0%; *P =* 0.0092).

#### Metazoa

In soil samples, Annelida and Rotifera were among the most dominant taxa across both systems, with higher (*P =* 0.0143) relative abundances of Annelida in organic soils (37.3% ± 14.1) than in conventional ones at time 3 (1.2% ± 0.6) and Rotifera (*P =* 0.05) at time 1 (16.4% ± 5.8 vs. 4.3% ± 1.8). In soil, Platyhelminthes (Catenulida) were more abundant in organic systems, at time 2 (7.8% vs. 0.0%, *P =* 0.0186) and 3 (4.7% vs. 0.0%, *P =* 0.0073). Copepoda were higher in conventional soils at time 2 (16.0% vs. 0.4%) and in organic soils at time 3 (5.3% vs. 0.0%, *P =* 0.029). Insects peaked in conventional soils at time 1 (18.0% vs. 0.0%, *P =* 0.0935). Other groups [e.g. Gastrotricha, Nematoda (Enoplea, Chromadorea), Tardigrada] varied across time but without consistent trends (Fig. 4d).

In water, Copepoda dominated, especially in conventional systems at time 2 (80.6% vs. 9.2%, *P =* 0.0253). Rotifera were also higher in conventional water at time 3 (33.0% vs. 5.6%, *P =* 0.05). Gastrotricha were more abundant in organic water at time 2 (35.8% vs. 0.4%, *P =* 0.0219), as were Platyhelminthes (Catenulida 8.2% vs. 0.3%, *P =* 0.0471; Fig. 4d).

## Discussion

This study provides a comprehensive comparison of microbial and mesofauna communities in the soil and overlying floodwater of organic and conventional rice farming systems throughout a growing season in South Brazil. Despite being one of the largest rice growing regions of the world there have been few studies looking at the microbial communities within the soil in rice paddies in Brazil and even less in organic systems (Lopes et al. 2011, Serbent et al. 2021), with few studies looking at microbial communities in the flood water in Brazilian rice agriculture (Reche et al. 2016, Pittol et al. 2018). Several studies have looked at microbial communities within conventional and organic rice paddies in both South (Mishra et al. 2025) and South-East Asia (Suzuki et al. 2019, Kuo et al. 2024) showing clear distinctions in communities between farming systems. Although environmental conditions and management practices differ across these studies, many are conducted in broadly comparable systems characterized by flooded cultivation, the same crop species, and strong seasonal dynamics. However, it is important to note that direct comparisons of environmental drivers are limited in the present study, as physicochemical parameters were not measured. As such, similarities in observed community patterns should be interpreted in the context of shared system characteristics rather than assumed equivalence in underlying environmental conditions.

However, the majority of published studies on organic and conventional rice farming focus only on bacteria and fungi, without profiling the unicellular eukaryote and the metazoan mesofauna in soil and in floodwaters, such as phytoplankton, heterotrophic protists, and zooplankton community. By analyzing bacteria, unicellular eukaryotes, fungi, and Metazoa across two distinct environmental compartments and three time points, we revealed clear, consistent effects of farming practices on microbial diversity and composition. These effects varied by microbial group and environment, reflecting the complex interactions between management inputs, habitat conditions, and seasonal change.

### Impact of farming system on richness and diversity

Both richness and diversity patterns varied across microbial groups and environments. In soil, fungal richness and diversity had a strong trend towards being higher in conventional plots, whereas bacterial richness was more varied across samples. Unicellular eukaryotes also showed a strong trend to high richness in conventional soils compared to organic. These patterns are likely due to inorganic fertilizer inputs or increased heterogeneity from disturbance and have been consistently observed across land use types and in different agricultural systems, where more heavily managed soils often have higher microbial diversity (George et al. 2019, Köninger et al. 2023). In the case of microbial groups this is usually attributed to higher soil pH in more heavily managed agricultural systems (Griffiths et al. 2011). In rice systems, including those in southern Brazil, soil pH and related chemical properties are known to respond to fertilization and amendment regimes, even within predominantly acidic soils (Araújo et al. 2008, da Silva et al. 2015). Together, these factors may create a wider range of ecological niches, supporting greater microbial coexistence in conventionally managed soils.

In contrast, in water, particularly bacteria, unicellular eukaryotes, and metazoans had a consistent trend of higher richness and diversity in organic plots at early and late time points. These differences suggest that water column communities may be more sensitive to short-term environmental changes, and that high nutrient loads or chemical residues in conventional systems may suppress diversity by supporting blooms of dominant taxa. This pattern has been seen in phytoplankton counts in rice paddies under different nutrient regimes, with higher richness and diversity in water from fields with no additions (Liu et al. 2020). Although between organic and conventional systems in Brazil, this pattern in phytoplankton has not been observed previously (Cassol et al. 2022). In the conventional system, bacterial richness and diversity were significantly lower at the beginning of the season, likely reflecting an immediate impact of surface-level inputs such as herbicides or fertilizers. Over time, microbial diversity in the water increased, particularly in organic systems, suggesting a gradual reassembly of more functionally diverse communities under more stable environmental conditions (Hester et al. 2022).

Taken together, these results highlight a clear divergence between soil and water compartments. In soils, higher richness and diversity in conventional systems may be associated with disturbance and nutrient enrichment, both of which can promote microbial proliferation and niche diversification. Such patterns have been widely reported across intensively managed agricultural soils. In contrast, water communities exhibited higher richness and diversity in organic systems, suggesting that reduced chemical inputs and more stable conditions favour more even and diverse assemblages. These contrasting responses underscore the compartment-specific nature of microbial community dynamics and indicate that soil and water environments are likely governed by different, and potentially decoupled, ecological drivers under contrasting farming practices.

### Impact of farming system on community composition

Analysis of the community composition showed consistent and significant differences between organic and conventional systems, in both soil and water, and across all microbial and Metazoa groups. This is similar to patterns seen in other studies which look at comparisons between organic and conventional systems (Lopes et al. 2011, Suzuki et al. 2019, Charaslertrangsi et al. 2024). The sources of fertilizers for each system are highly different—which will lead to different microbial communities developing which able to use the different carbon, nitrogen and phosphorus substrates. The application of both pesticides and herbcides in conventional farming will have a clear impact upon the metazoa and algal communities.

Interestingly these differences were most pronounced in floodwater and among eukaryotic groups, particularly for unicellular eukaryotes and Metazoa, which has been rarely looked at. Within each farming system, microbial communities in soil also changed significantly over time, reflecting seasonal turnover and crop development, which has been observed in rice paddy fields previously (Liu et al. 2016). However, the timing and direction of these changes differed by system, suggesting that farming practices not only shape microbial composition but also influence how communities shift through time (Lopes et al. 2011). While communities in the conventional farming samples may be adapted to long-term chronic impacts of repeated fertilizer, herbicide and pesticide applications, it is also likely there are shorter term acute impacts from the point of application (Abdullah et al. 1997, Zhang et al. 2025).

### Farming system impacts on whole communities in the rice paddy ecosystem

This study demonstrates that rice farming practices affect microbial and Metazoa communities in both compartments and across taxonomic groups. While soil variables were not looked at, these organic systems tended to support more stable and even communities in the water, while conventional systems showed higher richness in soil but also greater fluctuation, likely linked to synthetic inputs and physical disturbance. While NPG concentrations were low in water and did not show significant variation over time, its presence in soil alongside other management inputs may contribute to shaping community structure. However, disentangling the effects of specific compounds from the broader influence of farming systems remains a challenge.

Overall, these findings demonstrate the value of a multi-group, multi-compartment approach to understanding microbial ecology in rice systems. Including unicellular eukaryotes and Metazoa, and analyzing both soil and water compartments separately, provides a more complete picture of how farming affects biodiversity of the whole ecosystem.

### NPG and AMPA in soil and water of traditional and organic rice farming systems

While our primary focus was on microbial communities, the chemical data confirmed that conventional farming had consistently higher concentrations of NPG and its metabolite AMPA in both soil and water, particularly within early in the season in floodwater. This suggests that, shortly after application, the water column may be more exposed to acute inputs of NPG and AMPA. Levels were consistent with those reported previously in rice crop soil for NPG (Osten et al. 2025) and the levels detected in rice soils were around 35 times lower than for other crop types in Brazil (da Silva et al. 2021) which is consistent with other studies in the Americas (Osten et al. 2025). However, those studies may have very different soil and environmental conditions to this present study. As rice is a flooded crop, it is likely that dissolution to the water reduces the chronic pollution and buildups in soils, although the removal of that water or flooding during heavy rain may have implications for transfer of NPG and AMPA outside the fields and into the water courses. In the present study, no measurements were conducted in adjacent water bodies; therefore, this interpretation is based on the physicochemical properties of these compounds and previous literature describing their mobility in aquatic agricultural systems. Organic farming samples showed minimal to undetectable levels, consistent with the absence of any recent chemical inputs. These findings support the distinction in management regimes and provide important context for interpreting downstream biological responses. However, due to the complexity of the system and overlapping factors such as tillage, nutrient inputs, additional herbicide and pesticide, additional organic matter, flooding, and redox conditions, it is not possible to isolate the specific effects of glyphosate or AMPA on microbial communities from this dataset alone.

### Impacts of farming system on taxonomic composition

Bacterial communities responded clearly to farming system and time, particularly in soil. Acidobacteriae were consistently enriched in organic soils, which may reflect their adaptation to lower nutrient conditions and more complex carbon sources probably because of organic matter amendments to the soil, as suggested by previous studies, although these parameters were not directly measured in the present study (Kielak et al. 2016). In rice crop rhizosphere, Acidobacteriae are typical members of the community (Huang et al. 2020) and in other studies they have been shown previously to respond to organic amendments to soils in rice paddies (e.g. Tang et al. 2022). In contrast, Alphaproteobacteria showed an early-season peak in conventional soils, which may reflect their rapid response to increased nutrient availability and disturbance. Members of this group are often considered copiotrophic and metabolically versatile, allowing them to quickly exploit transient resource pulses. In addition, the degradation of glyphosate can release bioavailable phosphorus and carbon, potentially creating short-term nutrient enrichment that favors fast-growing taxa. Some Alphaproteobacteria species have also been reported to tolerate or degrade xenobiotic compounds, which may further contribute to their increased abundance following herbicide application (Zhu et al. 2024, Mishra et al. 2025). In conventional farming, fertilization itself can quickly avail significant amounts of resources to trigger populational peaks (Yin et al. 2025). Although some groups, such as Actinobacteria, were abundant across both systems, their temporal dynamics differed, with conventional soils showing greater fluctuation. These results suggest that soil bacterial communities are shaped by both resource availability and disturbance, with farming practice influencing the balance between community stability and turnover (Kuo et al. 2024). Few studies have looked at floodwater communities, but the dominance of Alphaproteobacteria and Actinobacteria is consistent with studies conducted in rice paddy systems in Brazil (Pittol et al. 2018).

Unicellular eukaryotes are important members of the rice paddy soil and water communities, and significantly impact bacteria through grazing in these systems (Murase et al. 2006, Asiloglu et al. 2021, Fujino et al. 2023, Murase and Asiloglu 2024). The present study has revealed a high diversity of unicellular eukaryotes in both soil and water, pointing out strong responses of these organisms to farming system, particularly in water. In organic systems, organisms in Zygnemophyceae and Cercozoa groups such as Filosa–Imbricatea and Filosa–Sarcomonadea were constantly more abundant, while conventional systems showed peaks in algal groups like Chrysophyceae and Cryptophyceae. In the case of herbicide inputs in these groups, a previous study did not observe differences in overall phytoplankton abundance because of herbicide application (Cassol et al. 2022). Thus, these shifts may reflect differences in nutrient regimes or disturbance effects (not directly measured in this study), with conventional inputs supporting blooms of opportunistic taxa at the expense of diversity (Murase et al. 2006, Murase and Asiloglu 2024). Heterotrophic protists such as Filosa-Sarcomonadea have been shown to respond to inputs of nitrogen fertilizers in paddy soils, frequently through increases in abundance, thus associated with higher bacterial prey availability (Bodur et al. 2024). Nitrogen enrichment can indirectly influence protist communities by stimulating bacterial growth, thereby favoring bacterivorous taxa and modifying community composition. Basal trophic level expansion can trigger responses throughout higher trophic levels, promoting a cascade effect able to increase community complexity. Similar patterns have been reported in other agricultural soils (Zhao et al. 2019). In relation to ciliates, Spirotrichia dominated the water component in this study, which is compatible with other reports that pointed out their high abundance and diversity in rice paddy fields. This result is probably due to their ability to survive in low oxygen environments (Schwarz and Frenzel 2003). These group-specific trends suggest that protists respond to both bottom-up effects, such as bacterial prey availability, and to environmental filters imposed by farming practices (Bodur et al. 2024).

Fungal community differences were less pronounced but still evident between farming systems. Sordariomycetes and Dothideomycetes dominated across most samples, but Rozellomycota varied more widely, showing higher abundance in conventional soils early in the season and in organic water samples later. Of interest is the dominance of the Rozellomycota, which are largely thought of as parasites of other fungi, algae, and protists (Corsaro et al. 2020). Their variable composition and diversity could likely be influenced by the availability of host organisms, as Rozellomycota are primarily parasitic and depend on the presence and dynamics of other microbial groups. Fungal taxonomic composition was similar to that detected in other studies and the Sordariomycetes, Rozellomycota, and Dothideomycetes have all been consistently part of rice paddy fungi communities (Zhang et al. 2019, Serbent et al. 2021, Pu et al. 2023, Kumar et al. 2024, Wang et al. 2024). Overall patterns in fungal taxonomic composition could reflect sensitivity to oxygen availability, nutrient inputs, and competition for resources. In rice paddies, fluctuating redox conditions influence fungal taxa with different oxygen tolerances, while differences between conventional and organic systems in nutrient availability may select for distinct functional groups. In addition, interactions with bacteria and protists can further shape fungal community structure through competition and resource dynamics (Zhang et al. 2019). In organic systems, the higher availability of organic matter, particularly during the early sampling time points, may have provided additional substrates for fungal growth, contributing to the observed differences in community composition between farming systems. Other fungal groups such as Endogonales and Pezizomycotina were more common in organic soils at specific time points, possibly due to differences in substrate availability or reduced chemical disturbance (Ma et al. 2022). Metazoan communities showed some of the most distinct differences between farming systems. Where studies have looked at Metazoa communities in rice paddies using conventional methods of profiling there were clear differences in the taxonomic composition between organic and conventional systems (Dalzochio et al. 2016). In this present study organic soils supported higher abundances of annelids and flatworms, especially later in the season, while rotifers and copepods were more common in conventional plots. The higher abundance of annelids is a consistent feature of organic management of most agricultural systems, and they thrive in the higher organic matter, lower nutrient, and lower disturbance management of these systems (Pelosi and Römbke 2016). Rotifers in our study sites would match findings from other studies focusing on Metazoa in rice paddy fields (e.g. Maiphae et al. 2023). In conventional soils this group had a trend towards higher abundance, and this may have been potentially stimulated by higher prey availability under nutrient-enriched conditions, as suggested in previous studies, although nutrient levels were not directly assessed in this study. Nonetheless, our findings were contrasting with results found by Romero et al. (2021) in a rice organic farming system. In addition, due to their small size, metabarcoding may have revealed a high diversity in this small metazoan group. Specifically, in rice paddies in the Americas macroinvertebrates have been shown to be important indicators in organic farming (Kumar et al. 2013). In water, conventional systems were dominated by copepods at mid-season, while organic plots supported a broader mix of taxa including rotifers, gastrotrichs and insects. These patterns may reflect cumulative differences in water quality, nutrient availability, and physical habitat, as described in previous studies, although these parameters were not directly measured here. As larger-bodied organisms with longer life cycles, metazoans may be especially sensitive to long-term management effects. At smaller scales the presence of nematodes in all soil plots is consistent with another study focused on mesofauna in rice paddies (Yang et al. 2020), where community composition was primarily affected by soil nutrient availability and C: N ratio.

In summary, our findings indicate that NPG might not be the most relevant cause of biodiversity disruptions when dealing with xenobiotics. AMPA was considered an agent which apparently caused more impact and long-lasting effects instead. Additional studies would be important to enhance understanding of long-term usage and potential accumulation of NPG and AMPA in soil in consecutive and multiple harvesting periods. The community structuring effects seemed to have caused the emergence of distinctive community subgroups as NPG and AMPA concentrations varied. Such prevalence probably denoted the aptitude of such subcommunities to prosper under transient predominant conditions at each of the established concentration intervals. NPG and AMPA presence, however, apparently did not cause the extinction of whole populations as observed by their successive emergence throughout the succession of crop stages including the variation in concentrations as time passed. Another important factor could be the availability of key chemical elements such as phosphorus and nitrogen as NPG was degraded, which could have exercised its share in the observed community structuring. Ultimately, our study highlighted the importance of a wide, multiparametric approach to understand effects xenobiotics may exert in microbial communities using different farming systems. It becomes clear the importance of assessing more than one organism category and attempting to understand the compounded effect different parameters might exert on the outcome. This work creates a baseline data on total communities of rice paddies in South Brazil, linking patterns to functional outcomes and long-term sustainability, particularly in relation to nutrient cycling, greenhouse gas emissions, and crop performance.

## Supplementary Material

fiag059_Supplemental_File

## Data Availability

Raw sequence reads (fastq) have been uploaded to the European nucleotide archive under the project accession number PRJEB89857.
