# Supplementary figures and images for: Impacts of conventional and organic farming practices on soil and aquatic microbial communities in rice (Oryza sativa) agricultural fields in Southern Brazil
Source: FEMS Microbiol Ecol. 2026 Jun 5;102(7):fiag059. doi: 10.1093/femsec/fiag059 (PMC13285872; doi:10.1093/femsec/fiag059)

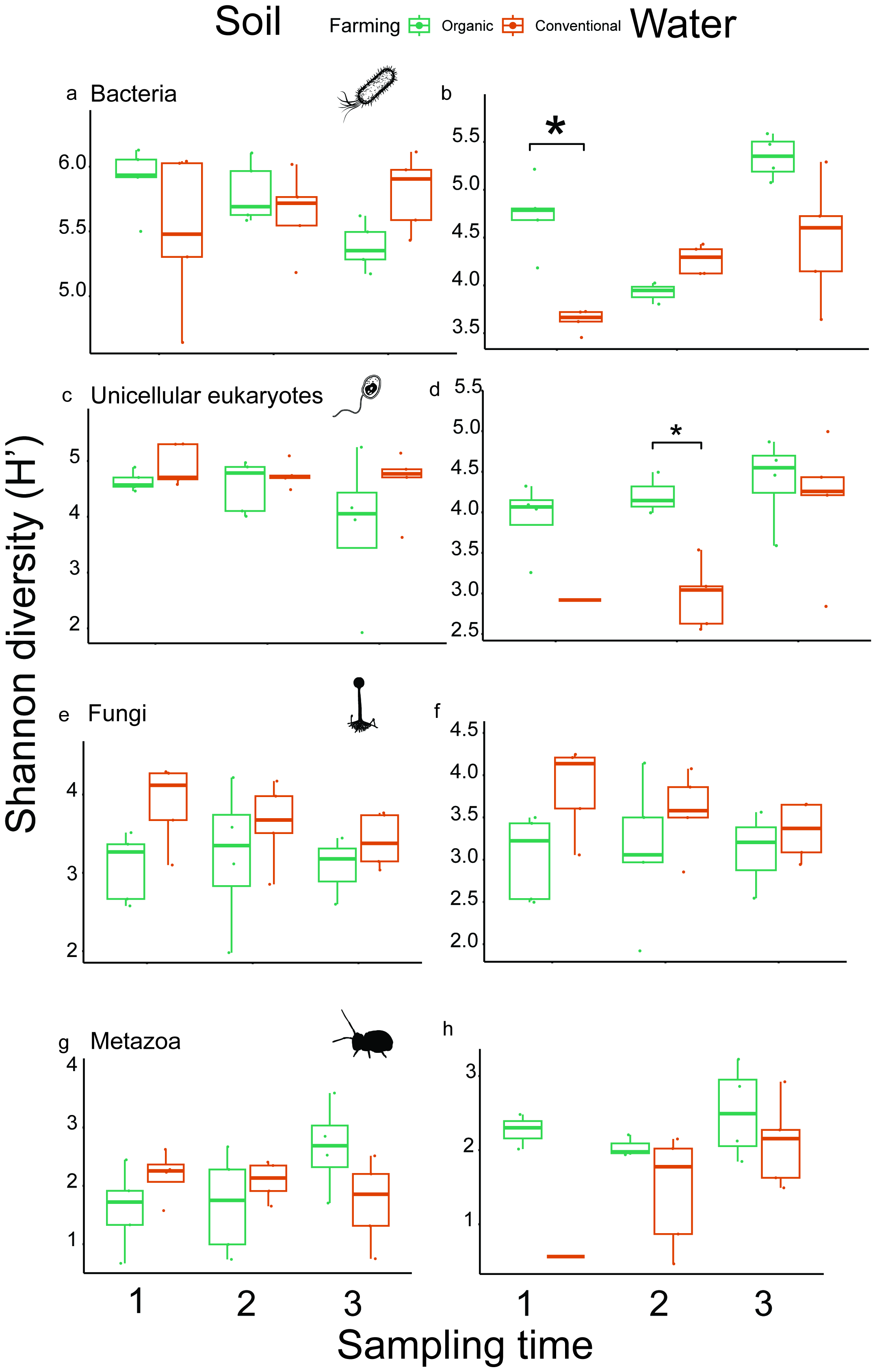

Supplement: fiag059_Supplemental_File [file fiag059_supplemental_file.zip › Supplementary figure 1.tif]
